# Diagnostic value of magnetic resonance imaging and magnetic resonance arthrography for assessing acetabular labral tears: A systematic review and meta-analysis

**DOI:** 10.1097/MD.0000000000032963

**Published:** 2023-03-03

**Authors:** Zhihao Huang, Wenyu Liu, Tianyu Li, Zhihao Liu, Pengfei Zhao

**Affiliations:** a School of Big Data and Fundamental Sciences, Shandong Institute of Petroleum and Chemical Technology, Dongying, China; b Department of Pharmacy, Weifang People’s Hospital, Weifang, China; c School of Education and Modern Communication, Shandong Institute of Petroleum and Chemical Technology, Dongying, China; d Department of Clinical Pharmacy, Weifang People’s Hospital, Weifang, China.

**Keywords:** acetabular labral tears, diagnosis, magnetic resonance arthrography, magnetic resonance imaging, meta-analysis

## Abstract

This study aimed to systematically evaluate the value of magnetic resonance imaging (MRI) and magnetic resonance arthrography (MRA) in the diagnosis of acetabular labral tears.

**Methods:**

Databases including PubMed, Embase, Cochrane Library, Web of Science, CBM, CNKI, WanFang Data, and VIP were electronically searched to collect relevant studies on magnetic resonance in the diagnosis of acetabular labral tears from inception to September 1, 2021. Two reviewers independently screened the literature, extracted data, and assessed the risk of bias in the included studies by using the Quality Assessment of Diagnostic Accuracy Studies 2 tool. RevMan 5.3, Meta Disc 1.4, and Stata SE 15.0 were used to investigate the diagnostic value of magnetic resonance in patients with acetabular labral tears.

**Results:**

A total of 29 articles were included, involving 1385 participants and 1367 hips. The results of the meta-analysis showed that the pooled sensitivity, pooled specificity, pooled positive likelihood ratio, pooled negative likelihood ratio, pooled diagnostic odds ratio, area under the curve of the summary receiver operating characteristic, and Q* of MRI for diagnosing acetabular labral tears were 0.77 (95% confidence interval [CI], 0.75–0.80), 0.74 (95% CI, 0.68–0.80), 2.19 (95% CI, 1.76–2.73), 0.48 (95% CI, 0.36–0.65), 4.86 (95% CI, 3.44–6.86), 0.75, and 0.69, respectively. The pooled sensitivity, pooled specificity, pooled positive likelihood ratio, pooled negative likelihood ratio, pooled diagnostic odds ratio, area under the curve of the summary receiver operating characteristic, and Q* of MRA for diagnosing acetabular labral tears were 0.87 (95% CI, 0.84–0.89), 0.64 (95% CI, 0.57–0.71), 2.23 (95% CI, 1.57–3.16), 0.21 (95% CI, 0.16–0.27), 10.47 (95% CI, 7.09–15.48), 0.89, and 0.82, respectively.

**Conclusion:**

MRI has high diagnostic efficacy for acetabular labral tears, and MRA has even higher diagnostic efficacy. Due to the limited quality and quantity of the included studies, the above results should be further validated.

## 1. Introduction

The acetabular labrum is a fibrocartilaginous ring attached to the edge of the acetabulum. It plays an important physiological role in ensuring wider coverage of the femoral head,^[1]^ reducing femoroacetabular joint contact pressure,^[2]^ and increasing the stability of the hip joint.^[3]^ The acetabular labrum increases the articular surface area by 22% and acetabular volume by 33% and is believed to create a seal in the hip joint.^[4]^ However, acetabular labral tear (ALT) destroys its physiological function, resulting in clinical symptoms such as hip pain and limited movement.^[5]^ ALT was first recognized as a pathological entity in 1957 when a bucket handle labral tear was discovered after an attempted reduction of a posterior hip dislocation.^[6]^ ALT can be associated with a variety of pathological conditions of the hip,^[7]^ and it is one of the most common causes of hip joint pain.^[8]^ It has been shown that hip and groin pain is caused by a labral tear in about 22% to 55% of patients.^[6]^ If not diagnosed and treated in time, the range of ALT increases and causes trauma,^[9]^ classic hip dysplasia,^[10,11]^ Legg–Calve–Perthes disease,^[12]^ and hip osteoarthritis.^[13]^

At present, the diagnostic methods of ALT mainly include magnetic resonance imaging (MRI), magnetic resonance arthrography (MRA), and arthroscopy. Arthroscopy is an invasive examination, which has the disadvantages of possible complications and high examination costs. Arthroscopy is generally carried out in the operation.^[14]^ Therefore, MR has become the first choice for the diagnosis of ALT. Because it is difficult to directly display the acetabular labral with computed tomography and X-ray, the detection rate of ALT was not high before MR examination is widely applied, and many patients were delayed the optimal treatment time due to lack of timely and correct diagnosis. With the widespread use of MR, the sensitivity (Sen) and specificity (Spe) of the diagnosis of ALT have been significantly improved. Many original studies have explored the value of MR in the diagnosis of ALT, but most of them were single diagnostic tests. In this study, a meta-analysis was conducted to comprehensively evaluate the value of MR in diagnosing ALT, to provide a basis for clinical diagnosis and scientific decision-making.

## 2. Methods

We adhered to the Preferred Reporting in Systematic Reviews and Meta-Analysis 2020 guidelines,^[15]^ and this review was registered in International Prospective Register of Systematic Reviews (registration number is CRD42021281868).

### 2.1. Eligibility criteria

The inclusion criteria were as follows: participants with suspected ALT who underwent MR before arthroscopy or surgery (not limited by age, race, and nationality); prospective or retrospective study design; direct or indirect availability of the results—true positive, false positive, false negative, and true negative.

The exclusion criteria were as follows: duplicate articles; articles with inconsistent research contents; non-English and non-Chinese articles; conference abstracts; case reports; and animal test.

Effect sizes included the pooled Sen, Spe, positive likelihood ratio (+LR), negative likelihood ratio (–LR), diagnosis odds ratio (DOR), summary receiver operating characteristics, area under the curve (AUC) of the summary receiver operating characteristic, and Q*.

### 2.2. Search strategy

A literature search was carried out by 2 independent reviewers. PubMed, Embase, The Cochrane Library, Web of Science, CBM, CNKI, WanFang Data, and VIP were explored from inception date to September 1, 2021.

### 2.3. Study selection and data extraction

Literature screening and data extraction were carried out independently by 2 reviewers. Different opinions were solved through discussion. Excel 2021 was used to extract data, mainly recording the first author, publication time, national research type, magnetic field intensity and examination method of MR, reference standard, age, gender, number of hips, and 4-fold data (true positive, false positive, false negative, and true negative).

### 2.4. Risk of bias assessment of the included studies

Two reviewers used the Quality Assessment of Diagnostic Accuracy Studies-2 tool to independently assess the risk of bias in the included studies.^[16]^ Each item was rated as “yes” (low bias or good applicability), “no” (high bias or poor applicability), or “unclear” (lack of relevant information or uncertain bias).

### 2.5. Statistical analysis

Review Management version 5.3 was used to assess the risk of bias in the included studies. Meta disc version 1.4 and Stata SE version 15.0 were used for meta-analysis. The correlation coefficient of Sen logarithm and (1 − Spe) logarithm was used to analyze whether there was a threshold effect. If the *P* value of the Spearman correlation coefficient was less than .05, it indicated that there was no threshold effect; otherwise, it indicated that there was a threshold effect. The heterogeneity of the meta-analysis results was tested by *χ*^2^ and *I*^2^. *χ*^2^ statistic with *P* < .1 or *I*^2^ > 50% indicated significant heterogeneity among the studies,^[17]^ which needed to be pooled by a random-effect model if the significant heterogeneity was not solved by meta-regression or subgroup analysis. Otherwise, a fixed-effect model was adopted. Sensitivity analysis was carried out by excluding the included studies one by one.^[18]^ The publication bias was detected by Deek funnel plot.^[19]^

## 3. Results

### 3.1. Literature search

**Figure 1.**
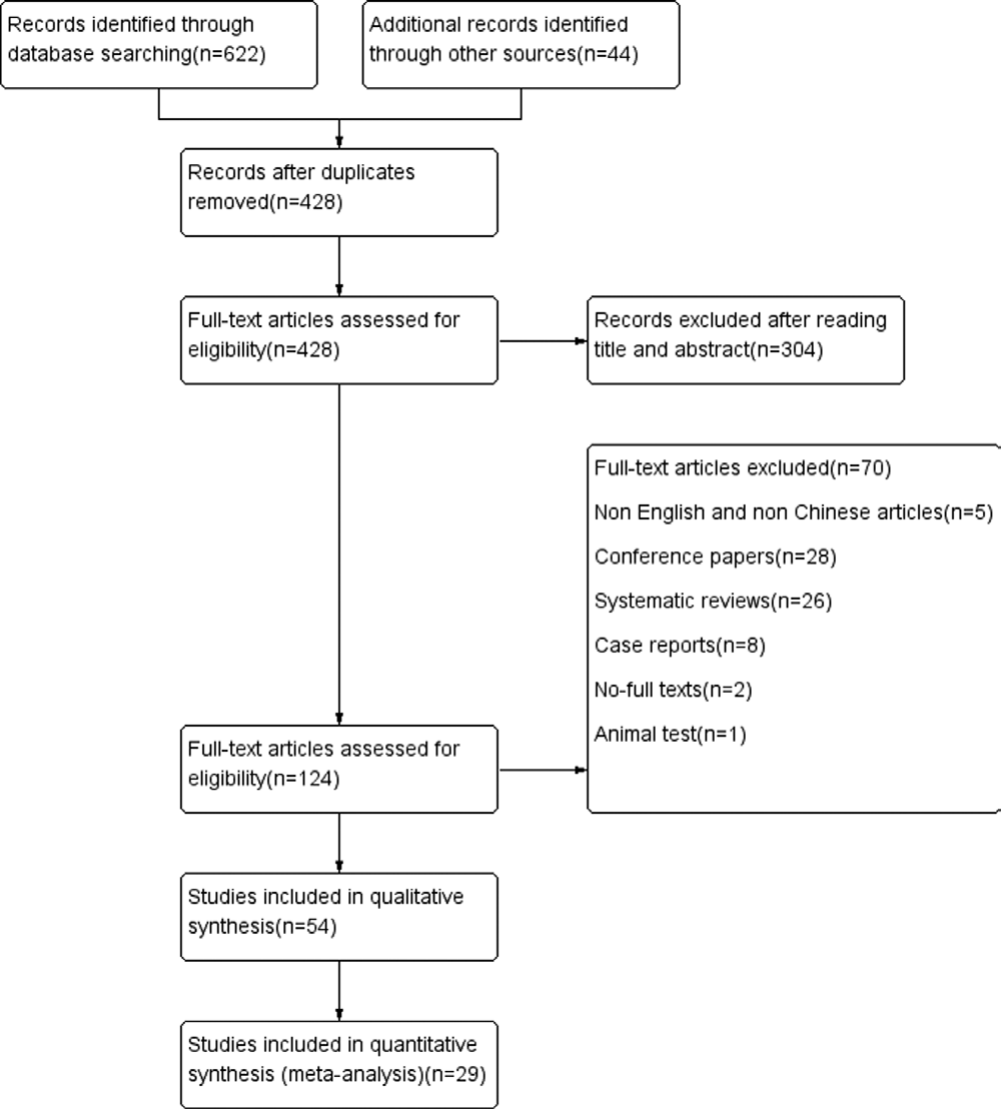
Flow diagram of the literature search and selection processes.

A total of 622 articles were identified by searching the databases. Additional 44 articles were identified during the screening of the reference sections of the included articles. The detailed information is shown in Method S1, Supplemental Digital Content, http://links.lww.com/MD/I463. After screening layer by layer, 29 articles were finally included.^[20–48]^ The process and the results of literature screening are shown in Figure 1.

### 3.2. Detailed information and risk of bias results

**Figure 2.**
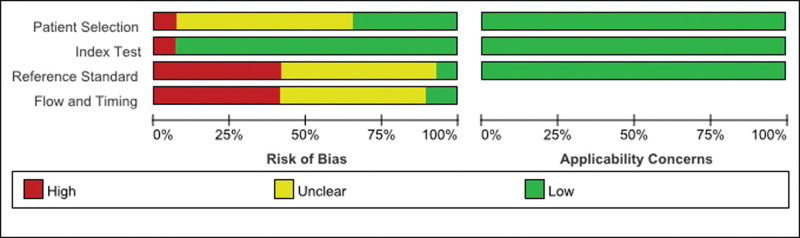
QUADAS-2 risk of bias and applicability concerns summary. QUADAS-2 = Quality Assessment of Diagnostic Accuracy Studies 2.

**Figure 3.**
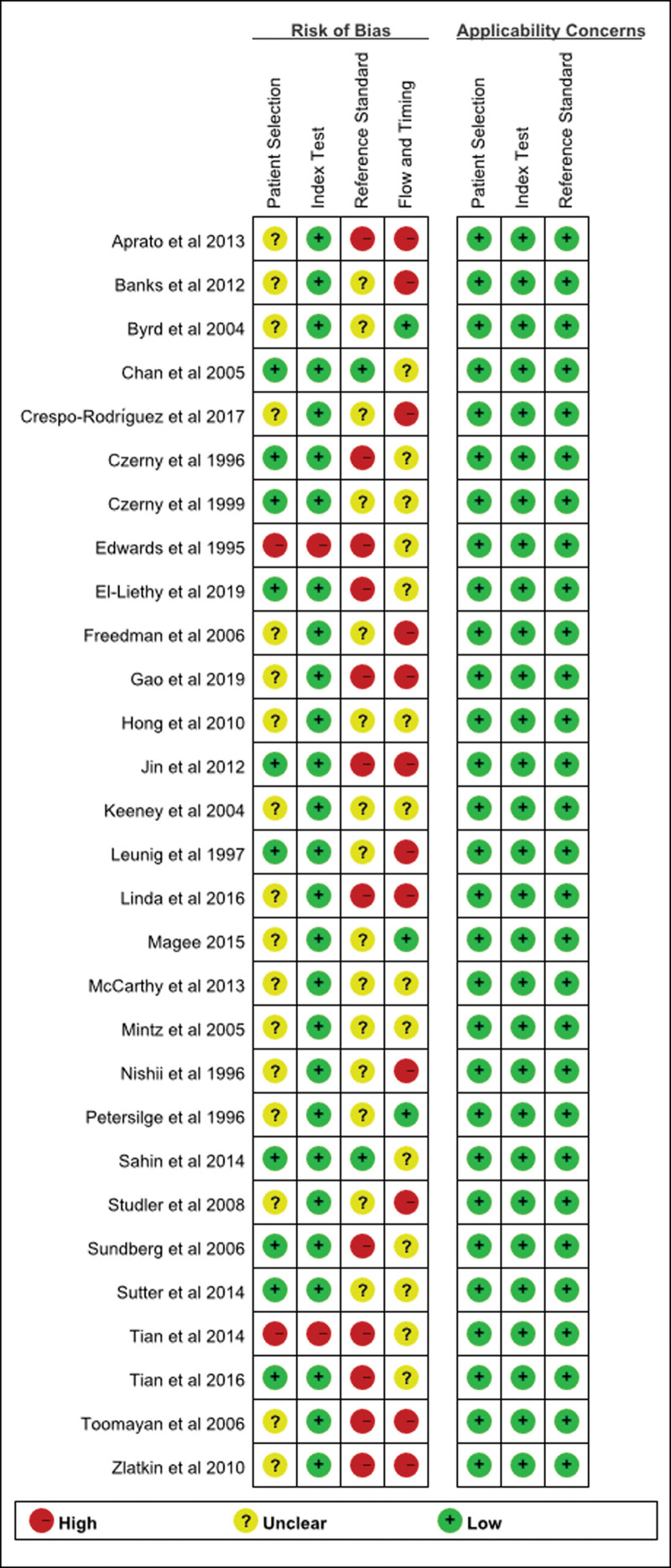
QUADAS-2 risk of bias and applicability concerns graph. QUADAS-2 = Quality Assessment of Diagnostic Accuracy Studies 2.

The detailed information of the included studies is shown in Table 1. The risk of biased results of the included studies is shown in Figures 2 and 3 and Table S1, Supplemental Digital Content, http://links.lww.com/MD/I464.

**Table 1:** Characteristics of included studies.

Study	Country	Type of study	Diagnostic method	Reference standard	Age (range)	Sample size (male/female)	Hip
Aprato et al 2013	Italy	Prospective	1.5T MRA	Surgical finds and Arthroscopy	24.0 (NA)	41 (24/17)	41
Banks et al 2012	England	Prospective	1.5T MRA	Arthroscopy	NA	66 (NA)	69
Byrd et al 2004	USA	Prospective	1.5T MRI and 1.5T MRA	Arthroscopy	NA	40 (NA)	40
Chan et al 2005	Taiwan	Prospective	1.5T MRA	Arthroscopy	41 (17–62)	30 (17/13)	17
Crespo-Rodríguez et al 2017	Spain	Retrospective	3.0T MRI and 1.5T MRA	Arthroscopy	42.5 (16–58)	50 (30/20)	50
Czerny et al 1996	Austria	Prospective	0.5T, 1.0T MRI and 0.5T, 1.0T MRA	Surgical findings	39 (14–68)	56 (15/41)	22
Czerny et al 1999	Austria	Prospective	0.5T and 1.0T MRA	Surgical findings	40 (14–67)	40 (9/31)	40
Edwards et al 1995	England	Prospective	1.5T MRI	Arthroscopy	35 (22–49)	23 (13/10)	23
El-Liethy et al 2019	Egypt	Prospective	1.5T MRA	Arthroscopy	31.9 (17–52)	31 (17/14)	31
Freedman et al 2006	USA	Prospective	1.5T MRA	Arthroscopy	37.1 (21–56)	24 (11/13)	24
Gao et al 2019	China	Retrospective	3.0T MRI	Arthroscopy	36.2 ± 9.6 (13–60)	195 (87/108)	195
Hong et al 2010	China	Prospective	1.5T MRA	Arthroscopy	50 (23–70)	15 (8/7)	14
Jin et al 2012	South Korea	Retrospective	3.0T MRA	Arthroscopy	43 (17–60)	16 (4/12)	16
Keeney et al 2004	USA	Retrospective	1.5T MRA	Arthroscopy	37.6 (NA)	101 (NA)	102
Leunig et al 1997	Switzerland	Prospective	1.5T MRA	Surgical findings	40 ± 2 (NA)	23 (9/14)	23
Linda et al 2016	Cananda	Retrospective	3.0T MRI	Arthroscopy	29 (13–45)	38 (25/13)	42
Magee 2015	USA	Retrospective	3.0T MRI and 3.0T MRA	Arthroscopy	34 (14–57)	43 (28/15)	43
McCarthy et al 2013	USA	Retrospective	1.5T MRA	Arthroscopy	36 (17–59)	62 (19/43)	70
Mintz et al 2005	USA	Retrospective	1.5T MRI	Arthroscopy	38.5 (15–74)	92 (34/58)	92
Nishii et al 1996	Japan	Prospective	1.5T MRA	surgical findings	39 (14–60)	18 (2/16)	19
Petersilge et al 1996	USA	Prospective	1.5T MRA	Surgical findings	38.4 ± 12.6 (27–72)	10 (5/5)	10
Sahin et al 2014	Turkey	Prospective	1.5T MRA	Surgical findings	34.1 ± 11.2 (19–52)	14 (3/11)	14
Studler et al 2008	Switzerland	Retrospective	1.5T MRA	Arthroscopy and arthrotomy	35 (15–68)	57 (21/36)	57
Sundberg et al 2006	USA	Prospective	3.0T MRI and 1.5T MRA	Arthroscopy	38 (NA)	8 (4/4)	8
Sutter et al 2014	Switzerland	Prospective	1.5T MRI and 1.5T MRA	Arthroscopy and surgical findings	31.8 (21–52)	28 (18/10)	28
Tian et al 2014	China	Retrospective	3.0T MRI and 3.0T MRA	Arthroscopy	35.1 ± 13.2 (NA)	90 (44/46)	90
Tian et al 2016	China	Retrospective	3.0T MRI	Arthroscopy	36 ± 13 (14–64)	122 (62/60)	122
Toomayan et al 2006	USA	Retrospective	1.5T MRI and 1.5T MRA	Arthroscopy	35 (14–63)	38 (21/27)	51
Zlatkin et al 2010	USA	Retrospective	1.5T MRI and 1.5T MRA	Arthroscopy	39 (19–68)	14 (5/9)	14

MRA = magnetic resonance arthrography, MRI = magnetic resonance imaging.

### 3.3. Meta-analysis of MRI

A total of 14 articles with 20 studies on 1246 hips were included (Table 2).

**Table 2:** Characteristics of MRI diagnostic tests.

Study name	Country	Magnetic field intensity	Hips	TP	FP	FN	TN
Byrd et al 2004	USA	1.5T	40	8	4	24	4
Crespo-Rodríguez et al 2017	Spain	3.0T	50	42	0	1	7
Czerny et al 1996	Austria	0.5T and 1.0T	22	6	0	14	2
Edwards et al 1995	England	1.5T	23	0	1	1	21
Gao et al 2019	China	3.0T	195	156	4	28	7
Linda et al 2016	Canada	3.0T	42	40	1	0	1
Magee 2015	USA	3.0T	43	38	1	4	0
Magee 2015	USA	3.0T	43	37	1	5	0
Mintz et al 2005	USA	1.5T	92	86	2	3	1
Mintz et al 2005	USA	1.5T	92	85	2	4	1
Sundberg et al 2006	USA	3.0T	8	5	2	0	1
Sutter et al 2014	Switzerland	1.5T	28	20	1	6	1
Sutter et al 2014	Switzerland	1.5T	28	23	1	3	1
Tian et al 2014	China	3.0T	90	36	7	23	24
Tian et al 2014	China	3.0T	90	39	8	20	23
Tian et al 2016	China	3.0T	122	53	9	34	26
Tian et al 2016	China	3.0T	122	56	9	31	26
Toomayan et al 2006	USA	1.5T	51	1	0	3	3
Toomayan et al 2006	USA	1.5T	51	1	0	11	2
Zlatkin et al 2010	USA	1.5T	14	11	0	2	1

FN = false negative, FP = false positive, MRI = magnetic resonance imaging, TN = true negative, TP = true positive.

### 3.4. Heterogeneity test

**Figure 4.**
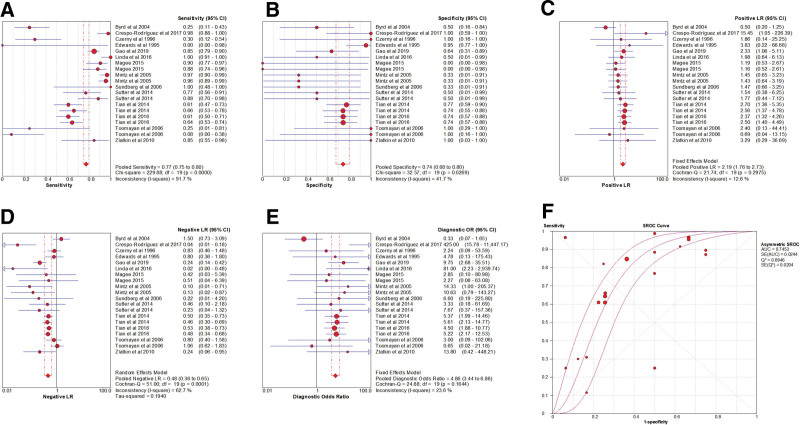
Forest plot of MRI for the diagnosis of ALT. Note: The subgraph of (A–F) refer to Sen, Spe, +LR, −LR, DOR, AUC, and Q*, respectively. ALT = acetabular labral tears, AUC = area under the curve, DOR = diagnosis odds ratio, −LR = negative likelihood ratio, +LR = positive likelihood ratio, MRI = magnetic resonance imaging, Sen = sensitivity, Spe = specificity.

Spearman correlation coefficient of Sen logarithm and (1 − Spe) logarithm was 0.570 (*P* = .009), indicating that there was a threshold effect in this study. The *I*^2^ of Sen and −LR was greater than 50%, and the effect sizes were pooled by the random-effect model. The *I*^2^ of Spe, +LR, and DOR was less than 50%, and the effect sizes were pooled by the fixed-effect model (Fig. 4).

### 3.5. Pooled effect sizes

The pooled effects sizes were as follows: Sen_(pooled)_ = 0.77 (95% confidence interval [CI], 0.75–0.80), Spe_(pooled)_ = 0.74 (95% CI, 0.68–0.80), +LR_(pooled)_ = 2.19 (95% CI, 1.76–2.73), −LR_(pooled)_ = 0.48 (95% CI, 0.36–0.65), DOR_(pooled)_ = 4.86(95% CI, 3.44–6.86), AUC = 0.75, and Q* = 0.69 (Fig. 4).

### 3.6. Meta-regression analysis

According to the study time, study country, and MRI magnetic field intensity, a meta-regression analysis was carried out. The results showed that study time was the main source of heterogeneity (*P* <.05).

### 3.7. Subgroup analysis

The variable with statistical significance in the meta-regression (study time) was analyzed in subgroups. The results were as follows:

### 3.8. Meta-analysis of subgroup_(Year: 1995–2009)_

Six articles with 8 studies on 379 hips were included (Table 2).

### 3.9. Heterogeneity test

Spearman correlation coefficient of Sen logarithm and (1 − Spe) logarithm was 0.739 (*P* = .036), indicating that there was a threshold effect in this subgroup study. The *I*^2^ of Sen and Spe was greater than 50%, and the effect sizes were pooled by the random-effect model. The *I*^2^ of +LR, −LR, and DOR was less than 50%, and the effect sizes were pooled by the fixed-effect model (Table 3).

**Table 3:** The heterogeneity of subgroup.

Group	n	Sen_(pooled)_		Spe_(pooled)_		+LR_(pooled)_		−LR_(pooled)_		DOR_(pooled)_	
		*I* ^2^	*P* value	*I* ^2^	*P* value	*I* ^2^	*P* value	*I* ^2^	*P* value	*I* ^2^	*P* value
Year											
1995–2009	8	95.2%	.000	64.9%	.006	0.0%	.629	42.5%	.095	28.9%	.198
2010–2019	12	86.9%	.000	12.2%	.325	0.0%	.589	47.7%	.033	0.0%	.487

DOR = diagnosis odds ratio, −LR = negative likelihood ratio, +LR = positive likelihood ratio, Sen = sensitivity, Spe = specificity.

### 3.10. Pooled effect sizes

The pooled effect sizes were as follows: Sen_(pooled)_ = 0.76 (95% CI, 0.70–0.81), Spe_(pooled)_ = 0.76 (95% CI, 0.61–0.87), +LR_(pooled)_ = 1.18 (95% CI, 0.79–1.77), −LR_(pooled)_ = 0.87 (95% CI, 0.65–1.16), DOR_(pooled)_ = 1.49 (95% CI, 0.63–3.56), AUC = 0.64, and Q* = 0.61 (Table 4).

**Table 4:** Results of subgroup analysis according to the characteristics of the study.

Subgroup	n	Sen_(pooled)_ (95% CI)	Spe_(pooled)_ (95% CI)	+LR_(pooled)_ (95% CI)	−LR_(pooled)_ (95% CI)	DOR_(pooled)_ (95% CI)	AUC	Q*
Year								
1995–2009	8	0.76 (0.70–0.81)	0.76 (0.61–0.87)	1.18 (0.79–1.77)	0.87 (0.65–1.16)	1.49 (0.63–3.56)	0.64	0.61
2010–2019	12	0.78 (0.75–0.81)	0.74 (0.66–0.80)	2.49 (1.93–3.21)	0.42 (0.36–0.50)	6.12 (4.22–8.88)	0.77	0.71

AUC = area under the curve, CI = confidence interval, DOR = diagnosis odds ratio, −LR = negative likelihood ratio, +LR = positive likelihood ratio, Sen = sensitivity, Spe = specificity.

### 3.11. Meta-analysis of subgroup_(Years: 2010–2019)_

Eight articles with 12 studies on 867 hips were included (Table 2).

### 3.12. Heterogeneity test

Spearman correlation coefficient of Sen logarithm and (1 − Spe) logarithm was 0.410 (*P* = .186), indicating that there was no threshold effect in this subgroup study. The *I*^2^ of Sen was greater than 50%, and the effect size was pooled by the random-effect model. The *I*^2^ of Spe, +LR, −LR, and DOR was less than 50%, and the effect sizes were pooled by the fixed-effect model (Table 3).

### 3.13. Pooled effect sizes

The pooled effect sizes were as follows: Sen_(pooled)_ = 0.78 (95% CI, 0.75–0.81), Spe_(pooled)_ = 0.74 (95% CI, 0.66–0.80), +LR_(pooled)_ = 2.49 (95% CI, 1.93–3.21), −LR_(pooled)_ = 0.42 (95% CI, 0.36–0.50), DOR_(pooled)_ = 6.12 (95% CI, 4.22–8.88), AUC = 0.77, and Q* = 0.71 (Table 4).

### 3.14. Sen analysis

**Figure 5.**
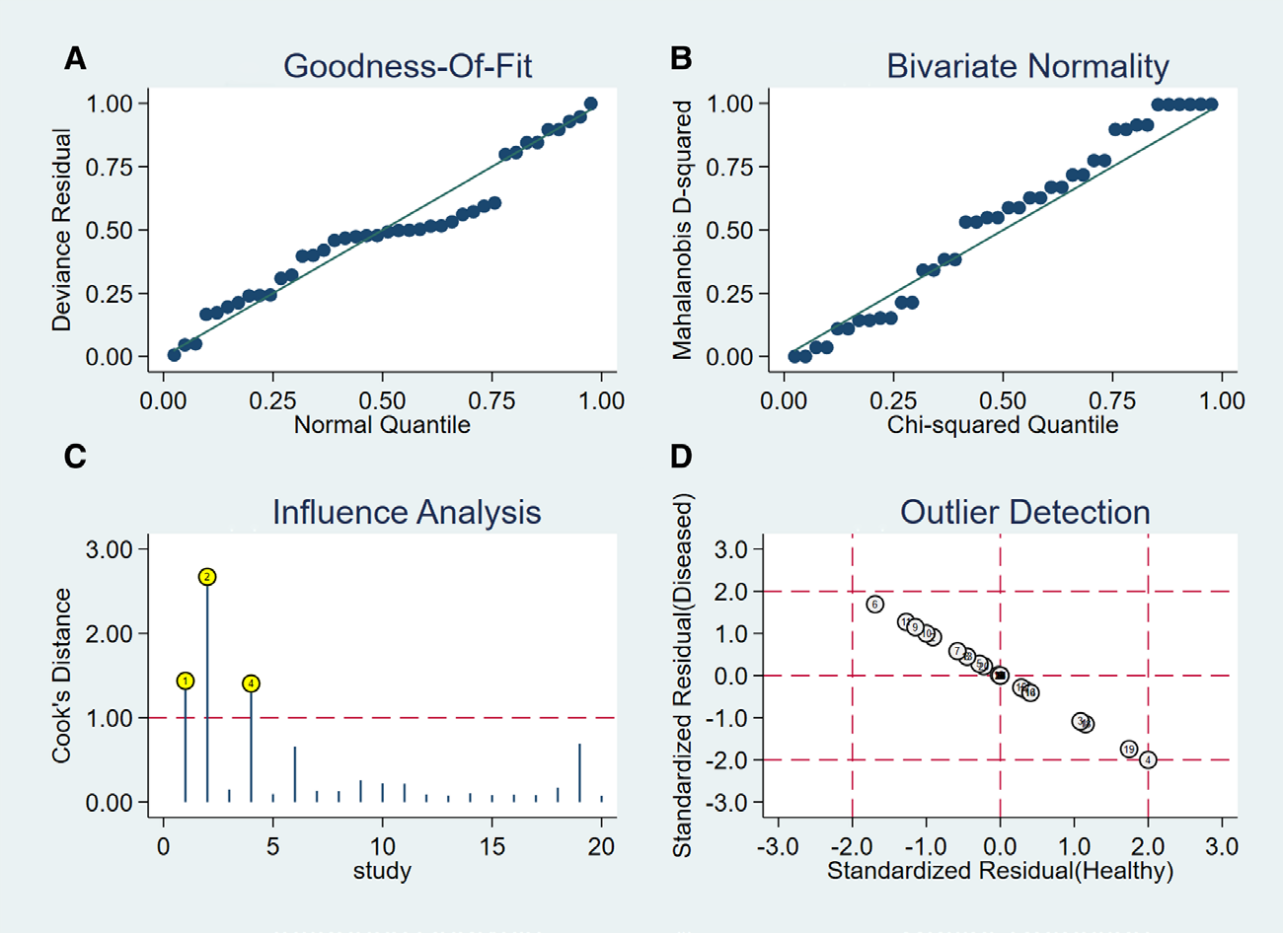
The sensitivity analysis of MRI. MRI = magnetic resonance imaging.

After excluding individual studies one by one, the remaining studies were pooled and analyzed again. The results showed that each study eliminated had little impact on the amount of pooled effect sizes, indicating that the results of this study were relatively stable and the reliability of the analysis results was high (Fig. 5).

### 3.15. Publication bias analysis

**Figure 6.**
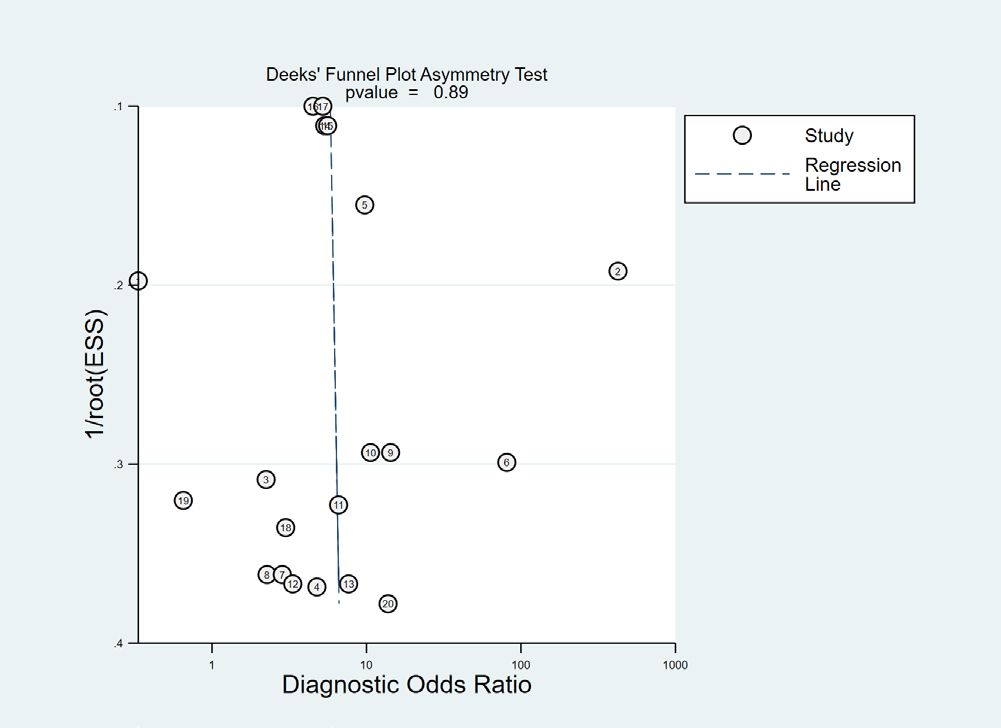
Funnel plot of MRI for the diagnosis of ALT. ALT = acetabular labral tears, MRI = magnetic resonance imaging.

Taking the inverse of the square root of effective sample size [1/root (ESS)] as the ordinate and DOR as the abscissa, the results of Deeks test showed that the *P* value of slope coefficient was 0.89, suggesting that there was no publication bias in the MRI examination method (Fig. 6).

### 3.16. Meta-analysis of MRA

A total of 24 articles with 27 studies on 942 hips were included (Table 5).

**Table 5:** Characteristics of MRA diagnostic tests.

Study name	Country	Magnetic field intensity	Hips	TP	FP	FN	TN
Aprato et al 2013	Italy	1.5T	41	31	1	3	6
Banks et al 2012	UK	1.5T	69	13	26	3	27
Byrd et al 2004	USA	1.5T	40	23	7	9	1
Chan et al 2005	Taiwan	1.5T	17	16	1	0	0
Crespo-Rodríguez et al 2017	Spain	1.5T	50	43	1	0	6
Czerny et al 1996	Austria	0.5T and 1.0T	22	18	0	2	2
Czerny et al 1999	Austria	0.5T and 1.0T	40	30	2	3	5
El-Liethy et al 2019	Egypt	1.5T	31	21	2	3	5
Freedman et al 2006	USA	1.5T	24	22	1	1	0
Hong et al 2010	China	1.5T	14	13	0	0	1
Jin et al 2012	South Korea	3.0T	16	10	1	1	4
Keeney et al 2004	USA	1.5T	102	66	5	27	4
Leunig et al 1997	Switzerland	1.5T	23	10	2	6	5
Magee 2015	USA	3.0T	43	39	1	3	0
Magee 2015	USA	3.0T	43	38	1	4	0
McCarthy et al 2013	USA	1.5T	70	49	3	11	7
Nishii et al 1996	Japan	1.5T	19	9	0	2	8
Petersilge et al 1996	USA	1.5T	10	8	0	0	1
Sahin et al 2014	Turkey	1.5T	14	10	2	0	2
Studler et al 2003	Switzerland	1.5T	57	43	6	1	7
Sundberg et al 2006	USA	1.5T	8	4	2	1	1
Sutter et al 2014	Switzerland	1.5T	28	22	0	4	2
Sutter et al 2014	Switzerland	1.5T	28	23	1	3	1
Tian et al 2014	China	3.0T	34	19	2	2	11
Tian et al 2014	China	3.0T	34	20	2	1	11
Toomayan et al 2006	USA	1.5T	51	22	0	2	6
Zlatkin et al 2010	USA	1.5T	14	13	1	0	0

FN = false negative, FP = false positive, MRA = magnetic resonance arthrography, TN = true negative, TP = true positive.

### 3.17. Heterogeneity test

**Figure 7.**
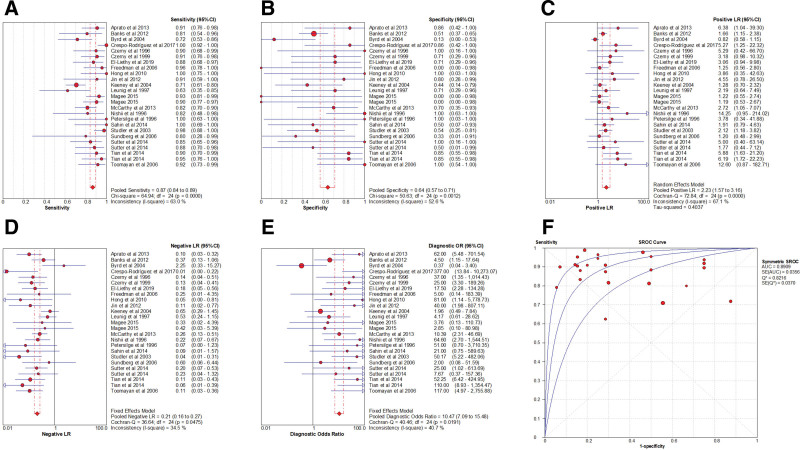
Forest plot of MRA for the diagnosis of ALT. Note: The subgraph of (A–F) refer to Sen, Spe, +LR, −LR, DOR, AUC, and Q*, respectively. ALT = acetabular labral tears, AUC = area under the curve, DOR = diagnosis odds ratio, −LR = negative likelihood ratio, +LR = positive likelihood ratio, MRA = magnetic resonance arthrography, Sen = sensitivity, Spe = specificity.

Spearman correlation coefficient of Sen logarithm and (1 − Spe) logarithm was − 0.153 (*P* = .465), indicating that there was no threshold effect in this study. The *I*^2^ of Sen, Spe, and +LR was greater than 50%, and the effect sizes were pooled by the random-effect model. The *I*^2^ of −LR and DOR was less than 50%, and the effect sizes were pooled by the fixed-effect model (Fig. 7).

### 3.18. Pooled effect sizes

The pooled effect sizes were as follows: Sen_(pooled)_ = 0.87 (95% CI, 0.84–0.89), Spe_(pooled)_ = 0.64 (95% CI, 0.57–0.71), +LR_(pooled)_ = 2.23 (95% CI, 1.57–3.16), −LR_(pooled)_ = 0.21 (95% CI, 0.16–0.27), DOR_(pooled)_ = 10.47 (95% CI, 7.09–15.48), AUC = 0.89, and Q* = 0.82 (Fig. 7).

### 3.19. Meta-regression analysis

According to the study time, study country, and MRI magnetic field intensity, a meta-regression analysis was carried out. The cause of heterogeneity was not found.

### 3.20. Sen analysis

**Figure 8.**
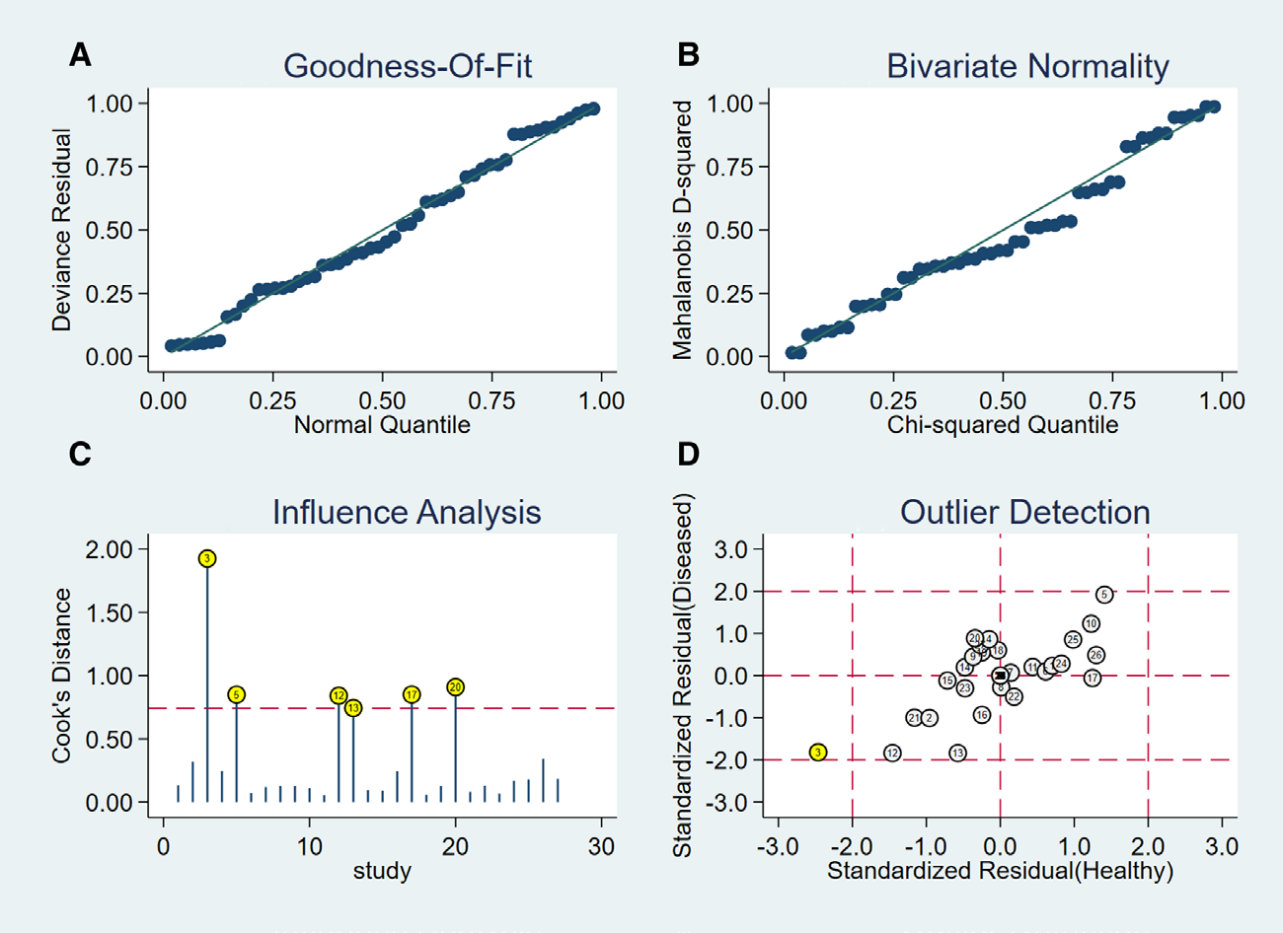
The sensitivity analysis of MRA. MRA = magnetic resonance arthrography.

After excluding individual studies one by one, the remaining studies were pooled and analyzed again. The results showed that each study eliminated had little impact on the amount of pooled effect sizes, indicating that the results of this study were relatively stable and the reliability of the analysis results was high (Fig. 8).

### 3.21. Publication bias analysis

**Figure 9.**
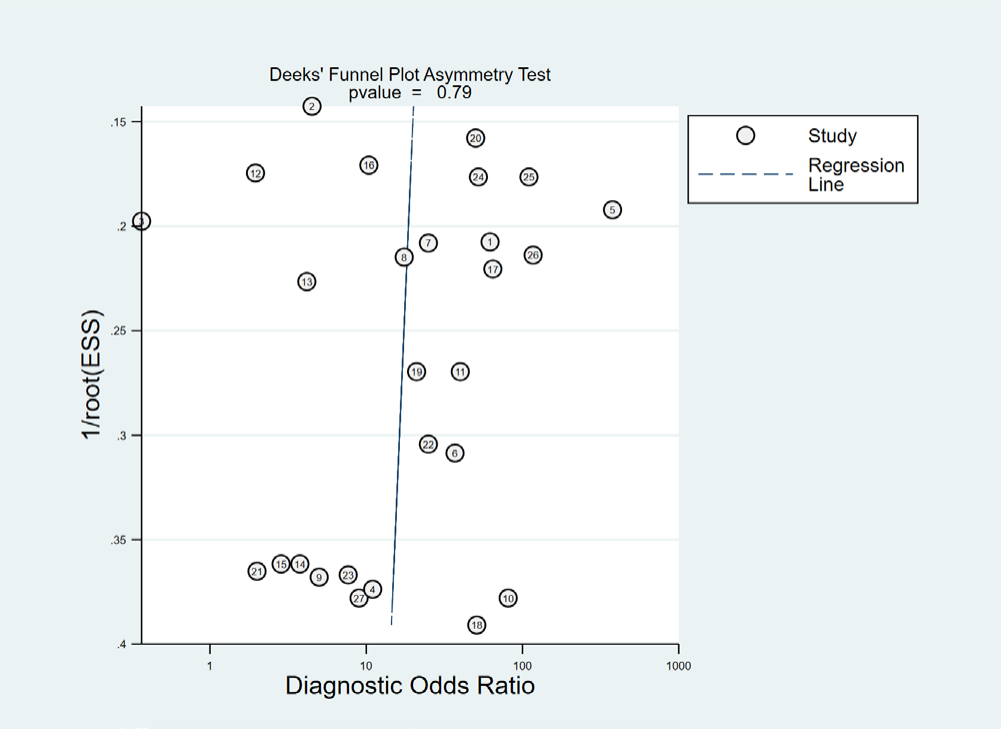
Funnel plot of MRA for the diagnosis of ALT. ALT = acetabular labral tears, MRA = magnetic resonance arthrography.

Taking the inverse of the square root of effective sample size [1/root (ESS)] as the ordinate and DOR as the abscissa, the results of Deeks test showed that the *P* value of slope coefficient was 0.79, suggesting that there was no publication bias in the MRI examination method (Fig. 9).

## 4. Discussion

The research quality of the included studies was assessed by the Quality Assessment of Diagnostic Accuracy Studies-2 tool. The results showed that the quality of the Applicability Concerns in 3 aspects, including Patient Selection, Index Test, and Reference Standard, was good. However, the Risk of Bias assessment in terms of Patient Selection, Reference Standard, and Flow and Timing was not satisfactory. The main reason is that the included studies did not provide answers to the following questions: “Was a consecutive or random sample of patients enrolled?”; “Were the reference standard results interpreted without knowledge of the results of the index tests?”; and “Was there an appropriate interval between index test and reference standard?” Additionally, some studies did not provide clear information about the following aspects: “The reference standard results interpreted with the knowledge of the results of the index tests”; “Not all patients receive the same reference standard”; and “Not all patients included in the analysis.”

By referring to the effect sizes of MRI and MRA, we found that MRI and MRA had high accuracy in diagnosing ALT, but the effect sizes Sen_(pooled)_ and DOR_(pooled)_ of MRA were higher than those of MRI, while the effect size −LR_(pooled)_ of MRA was lower than that of MRI. Considering that MRA has a higher diagnostic value, it has become the examination of choice for the evaluation of the acetabular labrum because of its excellent soft-tissue contrast and spatial resolution.^[49]^ When MRA is used, the injection of contrast media allows the joint capsule to expand and distinguish between the acetabular labral and the surrounding capsule tissue. The contrast media inserted into the acetabular labral also make the ALT more clearly displayed. Therefore, MRA has become the preferred imaging examination for the diagnosis of ALT. To explore the source of heterogeneity, this study also conducted meta-regression and subgroup analyses. The results of the subgroup analysis for different research years of MRI showed that the effect sizes +LR_(pooled)_ and DOR_(pooled)_ for the research years from 2010 to 2019 were higher than those for the research years from 1995 to 2009. The effect size −LR_(pooled)_ for the research years 2010 to 2019 was lower than that for the research years 1995 to 2009. This shows that in recent years, the rapid development of biotechnology has improved the diagnostic efficiency of MRI. According to the subgroup analysis of different MRI research year, we found that there was no threshold effect in the study after 2009, indicating that the diagnostic methods and evaluation criteria of MRI tend to be uniform after 2009.^[50]^

To improve the stability and reliability of the research results, during the implementation of this meta-analysis, 2 reviewers independently extracted the data and assessed the risk of bias. Strict inclusion and exclusion criteria were formulated during literature screening. Considering the differences between studies, meta-regression and subgroup analysis were carried out to find the source of heterogeneity. When the source of heterogeneity could not be found, the random-effect model was used to make the final results more reliable. This also makes our research more comprehensive than previous studies in terms of study time, study country and magnetic field intensity.^[51–53]^

Although meta-regression and subgroup analysis were carried out for the included studies, the reports of patients’ age, condition, and course of disease were incomplete, and there were certain differences in testing equipment and image analyst information, which might have also led to certain heterogeneity among the included studies. Moreover, the sample size of some studies was small, and the quality of some of the included studies was not very high. Finally, some studies regarded patients as research objects, while others regarded acetabular labrum as research objects, which also affect the results of this meta-analysis.

## 5. Conclusion

In this study, it was found that MR had a certain value in the diagnosis of ALT; in particular, MRA had higher diagnostic efficiency, and its application in the diagnosis of ALT was feasible in a clinical setting. However, due to the limitations of this study, the above conclusions still need to be further verified.

## Author contributions

**Conceptualization:** Zhihao Huang, Wenyu Liu, Tianyu Li.

**Formal analysis:** Zhihao Huang, Wenyu Liu, Pengfei Zhao.

**Investigation:** Wenyu Liu.

**Methodology:** Zhihao Huang, Zhihao Liu.

**Project administration:** Zhihao Huang.

**Supervision:** Zhihao Huang, Wenyu Liu.

**Visualization:** Zhihao Huang, Tianyu Li.

**Writing – original draft:** Zhihao Huang.

**Writing – review & editing:** Zhihao Huang, Wenyu Liu, Tianyu Li, Zhihao Liu, Pengfei Zhao.

## Supplementary Material




