# Supplementary material for: Diagnostic value of magnetic resonance imaging and magnetic resonance arthrography for assessing acetabular labral tears: A systematic review and meta-analysis
Source: Medicine (Baltimore). 2023 Mar 3;102(9):e32963. doi: 10.1097/MD.0000000000032963 (PMC9981430; doi:10.1097/MD.0000000000032963)
Supplement: Supplementary file 1 [file medi-102-e32963-s001.pdf]

Supplemental Digital Content

Supplemental Method 1 Electronic search strategies.

PubMed

#1 (((acetabular labral tears[Title/Abstract]) OR (acetabular labral[Title/Abstract])) OR (acetabula[Title/Abstract])) OR (acetabulum[Title/Abstract])

#2 (((((magnetic resonance imaging[Title/Abstract]) OR (magnetic resonance arthrography[Title/Abstract])) OR (magnetic resonance[Title/Abstract])) OR (MRI[Title/Abstract])) OR (MRA[Title/Abstract])) OR (MR[Title/Abstract])

#3 sensitiv*[Title/Abstract] OR sensitivity and specificity[MeSH Terms] OR (predictive[Title/Abstract] AND value*[Title/Abstract]) OR predictive value of tests[Title/Abstract] OR accuracy*[Title/Abstract]

#4 #1 AND #2 AND #3

Note:We searched 122 articles while using PubMed as the filter.

Embase

#1 'acetabular labral tears':ab,ti OR 'acetabular labra':ab,ti OR 'acetabula':ab,ti OR 'acetabulum':ab,ti

#2 'magnetic resonance imaging':ab,ti OR 'magnetic resonance arthrography':ab,ti 0R 'magnetic resonance':ab,ti OR 'MRI':ab,ti OR 'MRA':ab,ti OR 'MR':ab,ti

#3 'sensitiv':ab,ti OR 'sensitivity and specificity':ab,ti OR 'predictive':ab,ti OR 'predictive value of tests':ab,ti OR 'accuracy':ab,ti

#4 #1 AND #2 AND #3

Note:We searched 64 articles while using RefMan-(RIS) as the filter.

Cochrane Library

#1 (acetabular labral tears):ab,ti,kw OR (acetabular labra):ab,ti,kw OR (acetabula):ab,ti,kw OR (acetabulum):ab,ti,kw

#2 (magnetic resonance imaging):ab,ti,kw OR (magnetic resonance arthrography):ab,ti,kw OR (magnetic resonance):ab,ti,kw OR (MRI):ab,ti,kw OR (MRA):ab,ti,kw OR (MR):ab,ti,kw

#3 (sensitiv):ab,ti,kw OR (sensitivity and specificity):ab,ti,kw OR (predictive):ab,ti,kw OR (predictive value of tests):ab,ti,kw OR (accuracy):ab,ti,kw

#4 #1 and #2 and #3

Note:We searched 3 articles while using RefMan-(RIS) as the filter.

Web of Science

#1 TS=(acetabular labral tears or acetabular labra or acetabula or acetabulum)
#2 TS=(magnetic resonance imaging or magnetic resonance arthrography or magnetic resonance or MRI or MRA or MR)
#3 TS=(sensitiv or sensitivity and specificity or predictive or predictive value of tests or accuracy)
#4 #1 AND #2 AND #3
Note:We searched 178 articles while using Web of science as the filter.

CBM

1 "髋臼唇撕裂"[常用字段:智能] OR "髋臼唇"[常用字段:智能] OR "髋臼盂唇"[常用字段:智能]

2 "核磁共振"[常用字段:智能] OR "磁共振成像"[常用字段:智能] OR "MR 关节造影"[常用字段:智能] OR "MRI"[常用字段:智能] OR "MRA"[常用字段:智能] OR "MR"[常用字段:智能]

3 "诊断"[常用字段:智能]

4 1 AND 2 AND 3

Note:We searched 56 articles while using SinoMed as the filter.

CNIK

1 髋臼唇撕裂 OR 髋臼唇 OR 髋臼盂唇

2 核磁共振 OR 磁共振成像 OR MR 关节造影 OR MRI OR MRA OR MR

3 诊断

4 1 AND 2 AND 3

Note:We searched 69 articles while using NoteExpress as the filter.

WanFang Data

主题:(髋臼唇撕裂 or 髋臼唇 or 髋臼盂唇) and 主题:(核磁共振 or 磁共振成像 or MR 关节造影 or MRI or MRA or MR) and 主题:(诊断)

Note:We searched 109 articles while using NoteExpress as the filter.

VIP

#1 髋臼唇撕裂+髋臼唇+髋臼盂唇

#2 核磁共振+磁共振成像+MR 关节造影+MRI+MRA+MR

#3 诊断

#4 #1 与#2 与#3

Note:We searched 21 articles while using NoteExpress as the filter.
