# Supplementary material for: Diagnostic value of magnetic resonance imaging and magnetic resonance arthrography for assessing acetabular labral tears: A systematic review and meta-analysis
Source: Medicine (Baltimore). 2023 Mar 3;102(9):e32963. doi: 10.1097/MD.0000000000032963 (PMC9981430; doi:10.1097/MD.0000000000032963)
Supplement: Supplementary file 2 [file medi-102-e32963-s002.pdf]

Supplemental Digital Content

Supplemental Table 1 Details of quality assessment by the QUADAS-2 tool.

references	20	21	22	23	24	25	26	27	28	29	30	31	32	33	34	35	36	37	38	39	40	41	42	43	44	45	46	47	48
Was a consecutive or random sample of patients enrolled?	?	?	?	√	?	√	√	×	√	?	?	?	√	?	√	?	?	?	?	?	?	√	?	√	√	×	√	?	?
Was a case-control design avoided?	√	√	√	√	√	√	√	√	√	√	√	√	√	√	√	√	√	√	√	√	√	√	√	√	√	√	√	√	√
Did the study avoid inappropriate exclusions?	√	√	√	√	√	√	√	√	√	√	√	√	√	√	√	√	√	√	√	√	√	√	√	√	√	√	√	√	√
Are there concerns that the included patients and setting do not match the review question?	L	L	L	L	L	L	L	L	L	L	L	L	L	L	L	L	L	L	L	L	L	L	L	L	L	L	L	L	L
Were the index test results interpreted without knowledge of the results of the reference standard?	√	√	√	√	√	√	√	×	√	√	√	√	√	√	√	√	√	√	√	√	√	√	√	√	√	×	√	√	√
If a threshold was used, was it pre-specified?	√	√	√	√	√	√	√	√	√	√	√	√	√	√	√	√	√	√	√	√	√	√	√	√	√	√	√	√	√
Are there concerns that the index test, its conduct, or interpretation differ from the review question?	L	L	L	L	L	L	L	L	L	L	L	L	L	L	L	L	L	L	L	L	L	L	L	L	L	L	L	L	L
Is the reference standards likely to correctly classify the target condition?	√	√	√	√	√	√	√	√	√	√	√	√	√	√	√	√	√	√	√	√	√	√	√	√	√	√	√	√	√
Were the reference standard results interpreted without knowledge of the results of the index tests?	×	?	?	√	?	×	?	×	×	?	×	?	×	?	?	×	?	?	?	?	?	√	?	×	?	×	×	?	×
Are there concerns that the target condition as defined by the reference standard does not match the question?	L	L	L	L	L	L	L	L	L	L	L	L	L	L	L	L	L	L	L	L	L	L	L	L	L	L	L	L	L
Was there an appropriate interval between index test and reference standard?	√	?	√	?	√	?	?	?	?	√	?	?	?	?	?	?	√	?	?	?	√	?	√	?	?	?	?	√	?
Did all patients receive the same reference standard?	×	√	√	√	×	√	√	√	√	×	×	√	×	√	×	√	√	√	√	√	√	√	×	√	√	√	√	×	×
Were all patients included in the analysis?	√	×	√	√	√	√	√	√	√	√	×	√	√	√	√	×	√	√	√	×	√	√	√	√	√	√	√	√	×

Abbreviations**:**√=yes; ×=no; ?=unclear;L=low risk;U=unclear risk;H=high risk
